# Supplementary material for: Research on anti-rollover active control of sports utility vehicle with time-delay compensation function
Source: PLoS One. 2025 May 29;20(5):e0323996. doi: 10.1371/journal.pone.0323996 (PMC12121908; doi:10.1371/journal.pone.0323996)
Supplement: Attachment — Submitted filename: Response to Reviewers.docx [file pone.0323996.s002.docx]

**Responses to the comments from the editor and reviewers**

Dear editors and reviewers:

Thank you for your letter and for the reviewers' comments concerning our manuscript entitled “Research on anti-rollover active control of sports utility vehicle with time-delay compensation function”. The comments are all valuable and very helpful for revising and improving our paper, as well as the important guiding significance to our researches. We have studied comments carefully and have made correction which we hope meet with approval.

We have provided a point by point answer of each reviewer comment and highlighted (**red letters**) in the revised manuscript the passages that have been changed.

- **Reviewer 1**

*Overall, the study is well-designed and demonstrates promising results. The work is well-documented and reproducible. Addressing the given points would further improve it for publication by making it more concise and readable, here are some review comments for the paper*

**Response:** Thank you for your affirmation of our article.

1. *Discussion of how the approach could be validated further and integrated into practice scenarios.*

**Response:** Thank you very much for your good comments. We apologise for not taking this into account. It's our fault. After revision, we added the supplementary content at the end of the conclusion and marked it red. For your convenient, the added content was also listed as follow:

In this paper, the co-simulation of CarSim and MATLAB/Simulink verifies that the proposed ARCS effectively avoids the rollover of SUVs during cornering in both step steering and fishhook steering conditions. However, the real-world vehicle cornering situations are complex and variable, and the system’s actual performance has not been verified in the real world. Therefore, future work will focus on experimental testing to determine the reliability of the proposed ARCS in real-world applications. For example, the control algorithm can be embedded in the prototype vehicle’s electronic control unit (ECU), and the field experiment can be carried out in a controlled environment. In addition, the hardware in the loop (HIL) experiment can be used as an intermediate step to verify the real-time computing efficiency of the control system and the compatibility of automotive hardware. Integrating the system with automotive hardware and whether to compound safety standards are also tasks to be completed in the future.

2. *The discussion covers the key strengths and limitations. Relating back to how it builds on and advances prior work could highlight the significance.*

**Response:** Thank you very much for your good comments. Your comments made us realize that there are indeed shortcomings in this area and also gave us great inspiration. According to your comments, we made a revision at the end of the introduction and marked it in red. For your convenient, the added content was also listed as follow:

To sum up, most of the existing researches on vehicle rollover prevention methods mainly focus on the control of body roll angle, which is not the best choice among the various indicators of vehicle rollover prevention and does not consider the impact of time delay on the system control performance. In order to solve the limitations of previous studies, first of all, in selecting vehicle anti-rollover indicators, this paper selects *LTR*, which is universal and intuitive, as the vehicle anti-rollover indicator. Then, it establishes and verifies the *LTR* estimation model of the SUV. Then, considering the time-delay problem in the control system, the *LTR* is predicted based on the grey prediction model. Finally, the fuzzy controller is controlled in advance according to the predicted *LTR* to ensure the control performance. Therefore, the control method proposed in this paper can compensate for the time delay of the vehicle’s active ARCS, ensure control accuracy, and improve the vehicle’s active safety.

3. *The background of the proposed research should be further explained in detail. Please note that the up-to-date of references will contribute to the up-to-date of your manuscript. In this sense, in Introduction, authors should consider the following relevant papers: Quantized Iterative Learning Control of Communication Constrained System with the Encoding and Decoding Mechanism, Transactions of the Institute of Measurement and Control; ADP-Based Prescribed-Time Control for Nonlinear Time-Varying Delay Systems With Uncertain Parameters, IEEE Transactions on Automation Science and Engineering; Spatiotemporal fault estimation for switched nonlinear reaction–diffusion systems via adaptive iterative learning, International Journal of Adaptive Control and Signal Processing; By this rigorous literature analysis, your research's original results and divergence from usual methods will be revealed, strengthening the context in which your research is placed.:*

**Response:** Thank you for the above three papers of great reference value, which have a great effect on the improvement of our research. We have cited these three papers to enrich our literature review.

For your convenient, the added content was also listed as follow:

Tao et al. studied a quantitative iterative learning control based on a coding and decoding mechanism, which effectively solved the problem of data loss in a grid control system when the communication bandwidth was limited, and the load was high [22].

Zhang et al. proposed a prescribed time adaptive dynamic programming control method, which can optimize the steady-state performance of the nonlinear time-varying delay system and ensure that the system can accurately complete the task within a specific time range [23].

Peng et al. designed a fault estimator for spatiotemporal faults and used the iterative learning strategy to estimate the faults accurately [24].

4. *How should the proposed method's features be tuned for optimal performance?*

**Response:** Thank you very much for your good comments. It is true that this problem is not well reflected in the article, which is our thoughtlessness. In the last part of Article 4.2 prediction of the *LTR* and analysis of its accuracy, we made a supplementary description and marked it in red. For your convenient, the added content was also listed as follow:

Through iterative experiments, this paper determines the optimal modeling dimension (m) and prediction step (k). Specifically, the higher modeling dimension improves prediction stability but reduces responsiveness to sudden system changes. On the contrary, a smaller prediction step (e.g., *k*=2) enhances the prediction accuracy but cannot fully compensate for the longer time delay. Therefore, the trade-off between accuracy and responsiveness is considered when selecting the best parameters. The most important thing about ARCS proposed in this paper is grey prediction, and the most important thing about grey prediction is the modeling dimension and prediction step. Selecting the best modeling dimension and prediction step can make the performance of ARCS the best.

5. *All references must be accurate, and complete. Verify all cited papers, add missing volume/issue/page information, correct errors, and provide DOIs for unpublished references, which still have no volume and issue.*

**Response:** Thank you very much for your good comments. I'm sorry that we didn't take this into account. We have modified the references quoted in this article according to the standard reference format. For your convenience, all the references cited are listed at the end of this responses.

6. *The performance of the proposed method should be better analyzed, commented and visualized in the experimental section.*

**Response:** Thank you very much for your good comments. We are very sorry that we have not done well in this regard. Your comments have been of great help to us. According to your comments, we have made supplementary explanations and marked in red in 6.1 step steering condition and 6.2 fishhook steering condition respectively. For your convenient, the added content was also listed as follow:

a. In the last paragraph of 6.1 step steering condition:

Comprehensive Fig 8 and Fig 9 show that under the step steering condition, the vehicle rollover time is about 3.6s when the vehicle is without control, and the vehicle rollover time is about 4.2s when the vehicle is without predictive control. The vehicle can complete the steering smoothly when the vehicle is with predictive control. When the vehicle turned without control, all evaluation indicators were unstable, indicating that the vehicle would reverse soon after turning. When the vehicle turned without predictive control, although all evaluation indicators were better than without control, the vehicle still eventually rolled over due to the time delay in the controller’s response. However, with predictive control, the grey prediction system can accurately predict the vehicle’s driving status and potential risks, pre-calculate the possible time delay problem, and take corresponding compensation measures in time. In this way, the vehicle will trigger the ARCS when it is steering, and the system can carry out the control operation quickly and effectively through advanced preparation and precise prediction. It can accurately adjust the distribution of the vehicle’s center of gravity, optimize the contact state between the tires and the ground, and ensure that the vehicle is always stable during the steering process, thus enabling the vehicle to safely and smoothly complete the steering action, effectively avoiding traffic accidents triggered by rollover and other dangerous situations, and safeguarding the safety of the vehicle and the passengers.

b. In the last paragraph of 6.2 fishhook steering condition:

Combining Fig 11 and 12, it can be observed that in the fishhook steering condition, the vehicle rolled over at 3.4s without control and at 4.1s without predictive control. When the control is not applied, the vehicle exhibits obvious oversteer characteristics; the center of mass angle slip, yaw angle velocity, and body roll angle increase rapidly, the phase trajectory of the vehicle system is dispersed, and the SUV will roll over. In the case of not applying predictive control, although the addition of control can provide some control effect compared to not applying control, the presence of time delay makes control system’s response too slow, and the vehicle eventually rolls over. After the prediction mechanism is introduced into the vehicle control system, the time delay effect caused by signal processing, feedback delay, mechanical response, and other factors is significantly improved. Due to the addition of the prediction mechanism, the system can predict the dynamic changes of the vehicle in advance and timely adjust the suspension system, braking force distribution, power output and other key parameters of the vehicle at the critical moment. Through this precise control, the vehicle’s sideslip angle and body roll angle can be strictly controlled in a safe and stable area, and the lateral load transfer rate can also be maintained at a reasonable level of about 0.85. These ensure that the vehicle can maintain a good balance under lateral force and avoid insufficient tire grip or vehicle out of control caused by excessive load concentration. Finally, the vehicle remains stable throughout the driving process, successfully avoiding rollover accidents and improving driving safety and reliability.

7. *Have the authors considered employing other values in their research? What is the impact of the variables' sensitivity on the outcomes?*

**Response:** Thank you very much for your good comments. I am very sorry that we did not take this into account in the article. According to your comments, we have revised and marked the text in red. For your convenient, the added content was also listed as follow:

a. In paragraph 2, line 10 of 4.2 Prediction of the *LTR* and analysis of its accuracy:

In order to analyze the prediction accuracy further, the low threshold may lead to the premature start of the system and increase energy consumption. A higher threshold may cause the system to respond too late and increase the risk of rollover. Through experimental analysis and comparison of the results of various data in 0.7~0.9, it can be selected that the control system takes the lateral load transfer rate equal to 0.85 as the control threshold, that is, when the lateral load transfer rate reaches 0.85, the ARCS of SUV will start to work.

b. In paragraph 3, line 5 of 4.2 Prediction of the *LTR* and analysis of its accuracy:

Since the difference between the predicted value and the estimated value of *LTR* with modeling dimension outside [4,10] is too large, the accuracy of the modeling dimension in [4,10] is only analyzed in detail below.

c. In paragraph 5 of 4.2 Prediction of the *LTR* and analysis of its accuracy:

The prediction and sampling time significantly impact the model accuracy, while the control threshold selection directly affects the system’s response time and safety. Therefore, the sensitivity of these variables should be carefully considered when designing and optimizing the ARCS. Changes in these variables will directly affect the ARCS’s performance.

- **Reviewer 2**

1. *Abstract should be more about the problem studied, contributions and solutions, of course, in a short but informative way. Also, the abstract, like the title, should be able to stand alone and fully explain what your paper is about.*

**Response:** Thank you very much for your good comments. I am sorry that we have some deficiencies in the writing of the abstract of this article. Your comments are of great reference value to us. We have revised and marked red in the article. For your convenient, the revised content was also listed as follow:

The global incidence of traffic accidents caused by vehicle rollovers has exhibited a persistent upward trajectory in recent years. This paper proposes a novel rollover prevention control method incorporating time-delay compensation to address inherent latency issues in anti-rollover control systems (ARCS). First, structural parameters and dynamic theory establish a three-degree-of-freedom (3-DOF) dynamics model for a sport utility vehicle (SUV). Subsequently, a lateral load transfer ratio (*LTR*) estimation model is developed and validated under J-turn test conditions. A grey prediction model is then implemented to forecast *LTR* values in advance, compensating for system time delays. A two-dimensional fuzzy controller, utilizing error and error change rate as inputs, generates corrective yaw moment through differential braking to maintain vehicle stability. Co-simulation experiments conducted in CarSim and MATLAB/Simulink under typical driving scenarios demonstrate that the proposed method effectively mitigates ARCS time delays while preserving driving stability. The results suggest this approach provides both a practical solution for SUV rollover prevention and a conceptual advancement for vehicle active safety systems, showing strong potential for real-world implementation to reduce rollover risks and enhance road safety.

2. *In the abstract and conclusion, the contribution of this paper is not well presented. In the conclusion and abstract, highlight the novelty of the paper.*

**Response:** Thanks for your comments. Your comments have given us great inspiration in perfecting this article. According to your comments, we have revised and supplemented the article and marked it red. For your convenient, the modified content was also listed as follow:

a. In line 12 of the abstract, we add the contribution of the method proposed in this paper. As follows:

The results suggest this approach provides both a practical solution for SUV rollover prevention and a conceptual advancement for vehicle active safety systems, showing strong potential for real-world implementation to reduce rollover risks and enhance road safety.

b. In the conclusion part, we add the following content to reflect the novelty of the method proposed in this paper:

In order to prevent the SUV from rolling over when turning, this paper proposes a control system that can prevent the vehicle from rolling over. The system selects *LTR* as the rollover evaluation index. It uses the grey prediction model to predict the index in advance to compensate for the time delay in the system and to prevent the vehicle from rolling over in advance.

3. *The introduction is weak and should include the research question, the aim of the paper and the contribution.*

**Response:** Thanks for your comments. We are very sorry that it is our fault that this point is not done well. In the article, we have made corresponding revisions and marked in red. For your convenient, the modified content was also listed as follow:

a. As for the research question, we revised the first paragraph of the introduction as follows:

At present, road traffic accidents represent a significant cause of mortality. More than one million people are injured annually by road accidents [1]. The prevention of vehicle rollover is a critical factor in ensuring the safety of drivers and passengers. According to the U.S. National Road Safety Administration, although vehicle rollover accidents account for only 1.7% of all traffic accidents, they have a mortality rate as high as 33% [2]. The propensity for rollover accidents is particularly pronounced in sport utility vehicles (SUVs) due to their higher center of gravity and narrower wheelbase, rendering them more susceptible to rollover when steering to avoid obstacles or changing lanes. This elevated risk is substantiated by statistics showing that the rollover rate for SUVs is over nine times higher than typical sedans, with a correspondingly higher casualty rate [3]. Consequently, the development of rollover prevention technology has emerged as a prominent research focus within the domain of automobile active safety [4,5]. This paper mainly studies how to compensate for the time-delay phenomenon in the vehicle anti-rollover control system (ARCS) and provides a new method for improving it.

b. As for the purpose of this article, we have explained the compensation in the fourth paragraph of the introduction as follows:

To sum up, most of the existing researches on vehicle rollover prevention methods mainly focus on the control of body roll angle, which is not the best choice among the various indicators of vehicle rollover prevention and does not consider the impact of time delay on the system control performance. In order to solve the limitations of previous studies, first of all, in selecting vehicle anti-rollover indicators, this paper selects *LTR*, which is universal and intuitive, as the vehicle anti-rollover indicator. Then, it establishes and verifies the *LTR* estimation model of the SUV. Then, considering the time-delay problem in the control system, the *LTR* is predicted based on the grey prediction model. Finally, the fuzzy controller is controlled in advance according to the predicted *LTR* to ensure the control performance.

c. As for the contribution of this article, the ninth line of the fourth paragraph of the introduction is supplemented as follows:

Therefore, the control method proposed in this paper can compensate for the time delay of the vehicle’s active ARCS, ensure control accuracy, and improve the vehicle’s active safety.

4. *In related work…. Many researches work on this idea. What is really the novelty as compared to other studies? What is the new and the difference between the previous works and present work?*

**Response:** Thank you very much for your good comments. On this issue, we made a supplementary explanation in the last paragraph of the introduction. For your convenient, the added content was also listed as follow:

To sum up, most of the existing researches on vehicle rollover prevention methods mainly focus on the control of body roll angle, which is not the best choice among the various indicators of vehicle rollover prevention and does not consider the impact of time delay on the system control performance. In order to solve the limitations of previous studies, first of all, in selecting vehicle anti-rollover indicators, this paper selects *LTR*, which is universal and intuitive, as the vehicle anti-rollover indicator. Then, it establishes and verifies the *LTR* estimation model of the SUV. Then, considering the time-delay problem in the control system, the *LTR* is predicted based on the grey prediction model. Finally, the fuzzy controller is controlled in advance according to the predicted *LTR* to ensure the control performance. Therefore, the control method proposed in this paper can compensate for the time delay of the vehicle’s active ARCS, ensure control accuracy, and improve the vehicle’s active safety.

5. *What about last updating in this topic and new references from 2019-2025? The survey of existing literature is not sufficient. It would useful to include in the Introduction of the paper some discussion on other possible real applications of the obtained results.*

**Response:** Thank you very much for your good comments. Your comments are of great help to the improvement of our article. For this, we have also added some recent references for 2019-2025. For your convenient, the added content was also listed as follow:

Wang et al. proposed an integrated control strategy of distributed electric vehicles based on particle filter state estimation to realize the integration of handling, lateral stability, rollover prevention and ride comfort of distributed wheel motor-driven electric vehicles [12]. Jing et al. proposed a new integrated multi-objective control strategy for electric vehicles using multiple actuators to ensure the vehicle’s anti-roll and lateral stability [13]. Liu et al. proposed a 3-D Path Planning System Considering rollover and path length, which can plan a shorter path based on suppressing the roll angle of autonomous vehicles [14].

Tao et al. studied a quantitative iterative learning control based on a coding and decoding mechanism, which effectively solved the problem of data loss in a grid control system when the communication bandwidth was limited, and the load was high [22]. Zhang et al. proposed a prescribed time adaptive dynamic programming control method, which can optimize the steady-state performance of the nonlinear time-varying delay system and ensure that the system can accurately complete the task within a specific time range [23]. Peng et al. designed a fault estimator for spatiotemporal faults and used the iterative learning strategy to estimate the faults accurately [24].

# References:

12.	Wang Q, Zhao Y, Lin F, Zhang C, Deng H. Integrated control for distributed in-wheel motor drive electric vehicle based on states estimation and nonlinear MPC. Proceedings of the Institution of Mechanical Engineers, Part D: Journal of Automobile Engineering. 2021;236: 893–906. doi:10.1177/09544070211030444

13.	Jeong Y, Kim W, Yim S. Model Predictive Control Based Path Tracking and Velocity Control with Rollover Prevention Function for Autonomous Electric Road Sweeper. Energies. 2022;15: 984. doi:10.3390/en15030984

14.	Liu Z, Yuan X, Li Z. 3-D path planning system for autonomous vehicle considering the rollover and path length. J Frankl Inst-Eng Appl Math. 2022;359: 5272–5287. doi:10.1016/j.jfranklin.2022.05.040

22.	Tao Y, Tao H, Zhuang Z, Stojanovic V, Paszke W. Quantized iterative learning control of communication-constrained systems with encoding and decoding mechanism. Transactions of the Institute of Measurement and Control. 2024;46: 1943–1954. doi:10.1177/01423312231225782

23.	Zhang Z, Zhang K, Xie X, Stojanovic V. ADP-Based Prescribed-Time Control for Nonlinear Time-Varying Delay Systems With Uncertain Parameters. IEEE Trans Automat Sci Eng. 2025;22: 3086–3096. doi:10.1109/TASE.2024.3389020

24.	Peng Z, Song X, Song S, Stojanovic V. Spatiotemporal fault estimation for switched nonlinear reaction–diffusion systems via adaptive iterative learning. Adaptive Control & Signal. 2024;38: 3473–3483. doi:10.1002/acs.3885

6. *Figures are not clear. Clear diagrams and figures are required for readers to have clear images. Improve the quality of figures for better visibility. It is blur that should be adjusted.*

**Response:** Thank you very much for your good comments. We are sorry that we have not done well in this regard. We have revised it. For your convenient, the modified content was also listed as follow:

**Fig 3. Comparison of estimated value and actual value of *LTR* in J-Turn test.**

**Fig 8. Changes to Vehicle Rating Indicators.**

**Fig 9. Phase transition diagram.**

**Fig 11.** **Changes to Vehicle Rating Indicators.**

**Fig 12. Phase transition diagram**.

7. *Weak conclusion. Conclusion should be more specific with improvement writing quality.* *A suggestion for future work should be added in the conclusion section.*

**Response:** Thank you for your comments. It is our fault to have such a deficiency in the writing of the conclusion. We are very sorry for this. We have made changes and marked in red. For your convenient, the modified content was also listed as follow:

In order to prevent the SUV from rolling over when turning, this paper proposes a control system that can prevent the vehicle from rolling over. The system selects *LTR* as the rollover evaluation index. It uses the grey prediction model to predict the index in advance to compensate for the time delay in the system and to prevent the vehicle from rolling over in advance. The specific conclusions are as follows:

1) According to the characteristics of SUVs, a 3-DOF dynamic model of SUV rollover is established, and the *LTR* is used as the evaluation standard of vehicle rollover. Then, an *LTR* estimation model is established. The relative estimation error of the model is less than 2.7% through the J-turn test, which shows that the model can accurately estimate the *LTR* of SUVs.

2) Aiming at the time-delay problem of ARCS, the grey prediction model predicts the LTR of SUVs. The experimental data show that the maximum relative prediction error of the SUV *LTR* prediction model is less than 3%, which proves that this model can accurately predict the *LTR* of SUVs in advance.

3) Following the proposed active anti-rollover control method with time delay compensation, numerical simulations were performed under two different steering conditions, namely step and fishhook, and the results showed that the vehicle without control and without predictive control rolled over under both conditions. In the case of predictive control, the grey prediction system predicts in advance. It compensates for the time delay of the control system so that the center of mass slip angle of the vehicle can be controlled in the stable region, the *LTR* is maintained at about 0.85, and the vehicle does not roll over. To sum up, the method proposed in this paper has a good effect on compensating the time-delay phenomenon of ARCS. It can maintain the rollover dynamic stability of SUVs and improve the anti-rollover capability of SUVs.

In this paper, the co-simulation of CarSim and MATLAB/Simulink verifies that the proposed ARCS effectively avoids the rollover of SUVs during cornering in both step steering and fishhook steering conditions. However, the real-world vehicle cornering situations are complex and variable, and the system’s actual performance has not been verified in the real world. Therefore, future work will focus on experimental testing to determine the reliability of the proposed ARCS in real-world applications. For example, the control algorithm can be embedded in the prototype vehicle’s electronic control unit (ECU), and the field experiment can be carried out in a controlled environment. In addition, the hardware in the loop (HIL) experiment can be used as an intermediate step to verify the real-time computing efficiency of the control system and the compatibility of automotive hardware. Integrating the system with automotive hardware and whether to compound safety standards are also tasks to be completed in the future.

8. *Rewrite the references according to journal template.*

**Response:** Thank you for your comments. I'm sorry that we didn't take this into account. We have modified the references quoted in this article according to the standard reference format. For your convenience, all the references cited are listed at the end of this responses.

9. *Please strictly follow the instructions to the format specified in the journal template for preparing the paper.*

**Response:** Thank you for your comments. We are sorry we didn't follow the required format. We have modified the article according to the standard format.

10. *The format and English writing of this paper should be improved. The paper needs language revision.*

**Response:** Thank you for your comments. I am sorry that we are not good at the language writing of the article. We have improved the language logic and grammar of the article.

# References:

1.	Yari A, Hassanzadeh H, Akhbari K, Motlagh ME, Rahmani K, Zarezadeh Y. Hospital preparedness assessment for road traffic accidents with mass casualties: a cross-sectional study in Kurdistan Province, Iran. BMC Emerg Med. 2024;24: 68. doi:10.1186/s12873-024-00981-4

2.	Khan IU, Vachal K. Factors affecting injury severity of single-vehicle rollover crashes in the United States. Traffic Injury Prevention. 2020;21: 66–71. doi:10.1080/15389588.2019.1696962

3.	Seyedi M, Jung S, Wekezer J, Kerrigan JR, Gepner B. Rollover crashworthiness analyses – an overview and state of the art. International Journal of Crashworthiness. 2020;25: 328–350. doi:10.1080/13588265.2019.1593290

4.	Nguyen TA. Rollover prevention by designing a new fuzzy set solution for automotive active stabilizer bars based on a complex dynamic model. Advances in Mechanical Engineering. 2024;16: 16878132241298186. doi:10.1177/16878132241298186

5.	Nguyen TA. Assessing the dependence of vehicle rollover on many factors based on new 4D graphs. Zhang L, editor. PLoS ONE. 2023;18: e0284018. doi:10.1371/journal.pone.0284018

6.	Zhao D, Gong M, Zhao D, Liu W, Chang J, Tang J. Integrated control of anti-rollover for high-mobility emergency rescue vehicles. Proceedings of the Institution of Mechanical Engineers, Part D: Journal of Automobile Engineering. 2024; 09544070241231065. doi:10.1177/09544070241231065

7.	Nguyen TA. An original fuzzy control approach for active anti-roll bars to prevent rollover. Iqbal J, editor. PLoS ONE. 2023;18: e0290409. doi:10.1371/journal.pone.0290409

8.	Zhu B, Piao Q, Zhao J, Guo L. Integrated chassis control for vehicle rollover prevention with neural network time-to-rollover warning metrics. Advances in Mechanical Engineering. 2016;8: 1687814016632679. doi:10.1177/1687814016632679

9.	Vu VT, Sename O, Dugard L, Gaspar P. Enhancing roll stability of heavy vehicle by LQR active anti-roll bar control using electronic servo-valve hydraulic actuators. Vehicle System Dynamics. 2017;55: 1405–1429. doi:10.1080/00423114.2017.1317822

10.	Vu VT, Vu NT. Effect of Suspension Roll Angle Sensors on the Performance of *H*_∞_ Active Antiroll Bar Control System of Trucks. Dewasme L, editor. Mathematical Problems in Engineering. 2023;2023: 2715787. doi:10.1155/2023/2715787

11.	Guo Y, Wang B, Tkachev A, Zhang N. An LQG Controller Based on Real System Identification for an Active Hydraulically Interconnected Suspension. Shao X, editor. Mathematical Problems in Engineering. 2020;2020: 1–10. doi:10.1155/2020/6669283

12.	Wang Q, Zhao Y, Lin F, Zhang C, Deng H. Integrated control for distributed in-wheel motor drive electric vehicle based on states estimation and nonlinear MPC. Proceedings of the Institution of Mechanical Engineers, Part D: Journal of Automobile Engineering. 2021;236: 893–906. doi:10.1177/09544070211030444

13.	Jeong Y, Kim W, Yim S. Model Predictive Control Based Path Tracking and Velocity Control with Rollover Prevention Function for Autonomous Electric Road Sweeper. Energies. 2022;15: 984. doi:10.3390/en15030984

14.	Liu Z, Yuan X, Li Z. 3-D path planning system for autonomous vehicle considering the rollover and path length. J Frankl Inst-Eng Appl Math. 2022;359: 5272–5287. doi:10.1016/j.jfranklin.2022.05.040

15.	Shin D, Woo S, Park M. Rollover Index for Rollover Mitigation Function of Intelligent Commercial Vehicle’s Electronic Stability Control. Electronics. 2021;10: 2605. doi:10.3390/electronics10212605

16.	Zheng X, Tan P, Yao H, Xiang Y, Zhou H. An improved satin bowerbird optimization-based error-tracking compensation algorithm for active control system considering large time delay. Structures. 2024;61: 106008. doi:10.1016/j.istruc.2024.106008

17.	Tian Z, Wang Y. Predictive control compensation for networked control system with time-delay. Proceedings of the Institution of Mechanical Engineers, Part I: Journal of Systems and Control Engineering. 2022;236: 107–124. doi:10.1177/09596518211018298

18.	Luo H, Hu Z. Stability analysis of sampled-data control systems with multiple time-varying delays. Journal of the Franklin Institute. 2020;357: 6615–6634. doi:10.1016/j.jfranklin.2020.04.021

19.	Subramanian M, Kokil P. Stabilisation of network-controlled aircraft pitch control system with time delay. Transactions of the Institute of Measurement and Control. 2022;44: 2475–2484. doi:10.1177/01423312221083756

20.	Liu H, Li D, Zuo Z, Zhong Y. Robust attitude control for quadrotors with input time delays. Control Engineering Practice. 2017;58: 142–149. doi:10.1016/j.conengprac.2016.10.006

21.	Wang J, Liu C. Stabilization of uncertain systems with Markovian modes of time delay and quantization density. IEEE/CAA J Autom Sinica. 2018;5: 463–470. doi:10.1109/JAS.2017.7510823

22.	Tao Y, Tao H, Zhuang Z, Stojanovic V, Paszke W. Quantized iterative learning control of communication-constrained systems with encoding and decoding mechanism. Transactions of the Institute of Measurement and Control. 2024;46: 1943–1954. doi:10.1177/01423312231225782

23.	Zhang Z, Zhang K, Xie X, Stojanovic V. ADP-Based Prescribed-Time Control for Nonlinear Time-Varying Delay Systems With Uncertain Parameters. IEEE Trans Automat Sci Eng. 2025;22: 3086–3096. doi:10.1109/TASE.2024.3389020

24.	Peng Z, Song X, Song S, Stojanovic V. Spatiotemporal fault estimation for switched nonlinear reaction–diffusion systems via adaptive iterative learning. Adaptive Control & Signal. 2024;38: 3473–3483. doi:10.1002/acs.3885

25.	Liang W, Ahmac E, Khan MA, Youn I. Integration of Active Tilting Control and Full-Wheel Steering Control System on Vehicle Lateral Performance. IntJ Automot Technol. 2021;22: 979–992. doi:10.1007/s12239-021-0088-1

26.	Zhang Z, Rahman AHA, Zulkarnain N, Zhang T. Enhancing Vehicle Lateral Stability: A DDPG-Based Active Anti-Roll Bar Control Strategy. IEEE Access. 2024;12: 153030–153043. doi:10.1109/ACCESS.2024.3480116

27.	Yixuan F, Huanhuan Z, Minjie Y, Hongchao W. Risk Assessment Model of Emergency Lane Change Behavior forIntelligent Vehicles. MENG. 2024;17: 208–221. doi:10.2174/0122127976290621240212054625

28.	Xie N. A summary of grey forecasting models. GS. 2022;12: 703–722. doi:10.1108/GS-06-2022-0066

29.	Yan S, Gong X, Zeng X. Damping autoregressive grey model and its application to the prediction of losses caused by meteorological disasters. GS. 2025 [cited 19 Jan 2025]. doi:10.1108/GS-07-2024-0089

30.	Hu Y-C, Wu G. Forecast combination with multivariate grey prediction for tourism demand forecasting. IDA. 2024; 1–14. doi:10.3233/IDA-230565

31.	Li S, Wu N. A new grey prediction model and its application in landslide displacement prediction. Chaos, Solitons & Fractals. 2021;147: 110969. doi:10.1016/j.chaos.2021.110969

32.	Meng F, Gu J, Wang L, Qin Z, Gao M, Chen J, et al. A quantitative model based on grey theory for sea surface temperature prediction. Front Environ Sci. 2022;10: 1014856. doi:10.3389/fenvs.2022.1014856

33.	Zeng B, Li S, Meng W, Zhang D. An improved gray prediction model for China’s beef consumption forecasting. Li P, editor. PLoS ONE. 2019;14: e0221333. doi:10.1371/journal.pone.0221333

34.	Gao C, Hu Z, Xiong Z, Su Q. Grey Prediction Evolution Algorithm Based on Accelerated Even Grey Model. IEEE Access. 2020;8: 107941–107957. doi:10.1109/ACCESS.2020.3001194

35.	Du H, Zhang N. Robust stability control of vehicle rollover subject to actuator time delay. Proceedings of the Institution of Mechanical Engineers, Part I: Journal of Systems and Control Engineering. 2008;222: 163–174. doi:10.1243/09596518JSCE471

36.	Abbasi SMM, Jalali A. Fuzzy tracking control of fuzzy linear dynamical systems. ISA Transactions. 2020;97: 102–115. doi:10.1016/j.isatra.2019.07.028

37.	Dehghan M, Fateh MM, Ghalehnoie M. A Fuzzy-Supervised Impedance Control for an Active Suspension System. J Vib Eng Technol. 2023;11: 3257–3266. doi:10.1007/s42417-022-00746-y

38.	Gedefaw EA, Abdissa CM, Lemma LN. An improved trajectory tracking control of quadcopter using a novel Sliding Mode Control with Fuzzy PID Surface. Iqbal J, editor. PLoS ONE. 2024;19: e0308997. doi:10.1371/journal.pone.0308997

39.	Wang G, Liu L, Meng Y, Gu Q, Bai G. Integrated Path Tracking Control of Steering and Differential Braking Based on Tire Force Distribution. Int J Control Autom Syst. 2022;20: 536–550. doi:10.1007/s12555-021-0117-x

40.	Zou S, Zhao W, Wang C, Liang W, Chen F. Tracking and Synchronization Control Strategy of Vehicle Dual-Motor Steer-by-Wire System via Active Disturbance Rejection Control. IEEE/ASME Trans Mechatron. 2023;28: 92–103. doi:10.1109/TMECH.2022.3191999

41.	Li B, Bei S. Research method of vehicle rollover mechanism under critical instability condition. Advances in Mechanical Engineering. 2019;11: 1687814018821218. doi:10.1177/1687814018821218

Once again, thank you very much for your good comments and suggestions.
